# Unrecognized Cause of Native Valve Infective Endocarditis Due to Staphylococcus aureus: Laser Hair Removal

**DOI:** 10.7759/cureus.62798

**Published:** 2024-06-20

**Authors:** Kazunori Seo, Yuji Okazaki, Kyungko Huh, Toshihisa Ichiba

**Affiliations:** 1 Emergency Medicine, Hiroshima City Hiroshima Citizens Hospital, Hiroshima, JPN; 2 General Medicine, Hiroshima City Hiroshima Citizens Hospital, Hiroshima, JPN; 3 Infection Control, Hiroshima City Hiroshima Citizens Hospital, Hiroshima, JPN

**Keywords:** methicillin-sensitive staphylococcus aureus, infective endocarditis, native valve, laser hair removal, bacteremia

## Abstract

Laser hair removal for esthetic purposes has commonly been performed worldwide. This procedure is considered to be safe and effective, and severe complications such as systemic bacterial infections have seldom been reported. We present a case of native valve infective endocarditis (IE) potentially associated with laser hair removal. A 32-year-old female with a history of childhood atopic dermatitis presented with fever and arthralgia. She had been receiving monthly total body laser hair removal treatments for nine months. Physical examination revealed numerous painful purpuras on her fingers and soles. Laboratory examinations revealed a positive troponin level, and a 12-lead electrocardiogram revealed ST-segment elevation in inferolateral leads. Transthoracic echocardiography revealed mild wall motion abnormalities from the mid-posterior wall to the apex and thickening of the anterior mitral valve leaflet. Blood cultures grew methicillin-susceptible *Staphylococcus aureus* (MSSA). Based on these findings, we made a diagnosis of native valve *Staphylococcal* IE and acute myocardial infarction due to septic embolism. Due to the progression of mitral valve destruction, she underwent mitral valve replacement surgery and received an eight-week course of antibiotics, leading to a successful recovery. This case highlights a potential association between laser hair removal and *Staphylococcal* IE. Laser hair removal may compromise the skin barrier, potentially allowing the entry of bacteria such as *S. aureus*. Increased awareness of this potential complication is necessary, especially in populations at high risk for IE. Further research is needed to investigate the link between laser hair removal and bacteremia, particularly in high-risk populations, to guide prevention strategies.

## Introduction

Infective endocarditis (IE) presents a significant challenge to clinicians due to the complexities involved in its diagnosis, treatment, and prevention. The most common pathogens are *Staphylococcus aureus* and *Streptococcus* species, with portals of entry for these bacteria including the skin, teeth, and intestinal tract, although the exact site of entry is sometimes not clear [1]. However, identifying the portal of entry is crucial for managing the disease and preventing its recurrence.

The practice of laser hair removal for esthetic purposes has gained global popularity [2]. Although rare complications arising from this procedure are predominantly perceived as being localized skin issues, systemic bacterial infections such as bacteremia have not been reported [3]. We present a case of native valve IE caused by *Staphylococcus aureus*, which might have been triggered by laser hair removal.

## Case presentation

**Figure 1:**
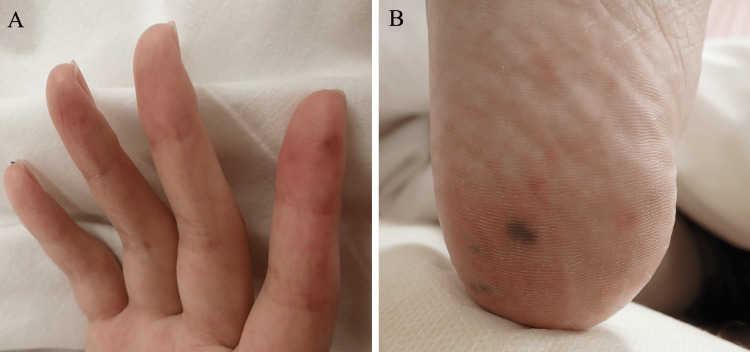
Physical examination. (A) Painful purpura was found on the right fourth finger. (B) Multiple painful purpuric lesions were found on the right toes.

A 32-year-old female presented to our emergency department with a complaint of fever and generalized arthralgia for four days in 2023. She had a history of atopic dermatitis that had resolved in childhood, and she had not used topical agents for approximately twenty years. She was not an intravenous drug user. For the past nine months, she has been receiving monthly laser hair removal treatments at a beauty clinic for total body hair removal. The most recent session was performed two weeks prior to the onset of her symptoms, and her entire body was exposed to the laser in that session. Each session lasted approximately one and a half hours, and topical steroids were applied after treatment. During the sessions, she did not receive any intravenous injections, such as a sedative agent. After receiving the treatment, she had experienced transient erythema for a few days. On examination, she was alert with a temperature of 37.6°C, blood pressure of 89/54 mmHg, heart rate of 111 beats/min, respiratory rate of 18 breaths/min, and oxygen saturation of 96%. No heart murmurs were detected. Although dry skin, pruritus, and eczema were not observed in her skin, numerous painful purpuras were noted on her fingers and soles (Figure 1).

Laboratory examinations showed an elevated C-reactive protein level and N-terminal pro-brain natriuretic peptide (NT-pro BNP) level, a decreased platelet count, and a positive troponin T level (Table 1).

**Table 1:** Laboratory examination. ALT: alanine transaminase; AST: aspartate transferase; γ-GTP: γ-glutamyl transpeptidase; ALP: alkaline phosphatase; LD: lactate dehydrogenase; NT-pro BNP: N-terminal pro-brain natriuretic peptide.

Test	Hospital admission	Reference range
White blood counts (x10^3^/μL)	5.9	3.3–8.6
Hemoglobin (g/dL)	12.6	11.6–14.8
Platelet (x10^4^/μL)	7.6	15.8–34.8
AST (U/L)	199	13–30
ALT (U/L)	136	10–42
γ-GTP (U/L)	38	13–64
ALP (U/L)	84	38–113
LD (U/L)	409	124–222
Blood urea nitrogen (mg/dL)	20	8–20
Creatinine (mg/dL)	0.8	0.65–1.07
Creatinine kinase (U/L)	951	59–248
C-reactive protein (mg/dL)	24.6	<0.14
Troponin T (ng/mL)	0.838	<0.1
NT-pro BNP (pg/mL)	4705	<125

**Figure 2:**
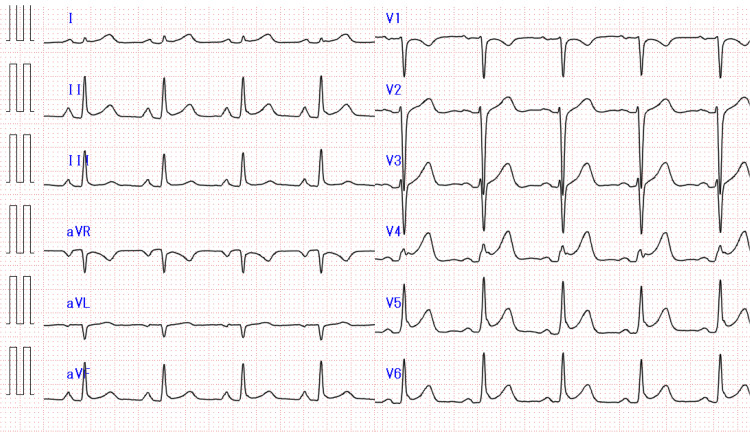
12-lead electrocardiogram. A 12-lead electrocardiogram showed ST-segment elevation in leads II, III, aVF, and V4 to V6.

A 12-lead electrocardiogram showed ST-segment elevation in leads II, III, aVF, and V4 to V6 (Figure 2).

Transthoracic echocardiography (TTE) revealed mild wall motion abnormalities from the mid-posterior wall to the apex and thickening of the anterior mitral valve leaflet, but no vegetations or mitral regurgitation were observed. We considered the possibility of IE and acute myocardial infarction due to coronary embolism caused by IE. After admission to the intensive care unit (ICU), we intravenously administered 2 g ceftriaxone after obtaining a blood culture.

**Figure 3:**
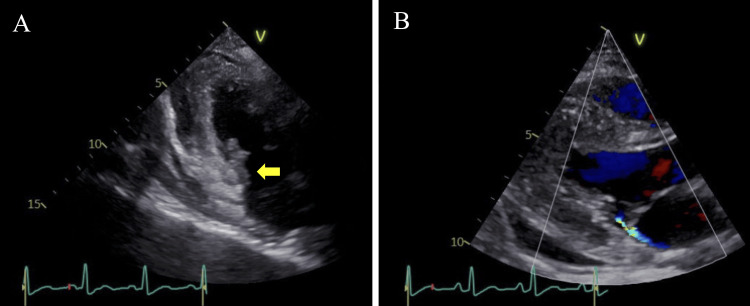
Transthoracic echocardiography. Transthoracic echocardiography showed an isoechoic area extending from the posterior commissure to the posterior papillary muscle (yellow arrow) (A), and a jet directed toward the posterior wall of the left atrium (B).

Twelve hours after admission, methicillin-susceptible *Staphylococcus aureus* (MSSA) was detected in the blood culture. Magnetic resonance imaging of the head revealed multiple small cerebral infarcts. Consequently, we diagnosed her with a native valve IE due to MSSA. She was treated with antibiotics and circulatory management using noradrenaline. On the second day, TTE showed an isoechoic area extending from the posterior commissure to the posterior papillary muscle, suggesting vegetation and mild mitral regurgitation (Figure 3).

On the seventh day, acute heart failure due to the progression of mitral valve destruction occurred, leading to artificial valve replacement surgery on the eighth day. MSSA was also detected in the resected mitral valve. She was extubated on the fourth postoperative day and transferred out of the ICU on the ninth postoperative day. She received antimicrobial therapy for a total of eight weeks and was discharged without any complications.

## Discussion

Laser hair removal may have a negative impact on the skin barrier, potentially leading to the development of IE. The treatment is preferred for esthetic purposes as well as for hirsutism or excess hair growth because it is considered to be non-invasive, safe, and effective [2,4]. The most common lasers are effective for hair removal because of their ability to target melanin in the hair shafts and follicles and penetrate to an appropriate dermal depth to selectively destroy the hair follicles [4]. Although there is slight pain during exposure to the laser, skin complications such as transient erythema are usually mild [3]. However, micro-inflammation around the hair follicles may permit bacterial invasion in the skin. In fact, skin infections due to a non-tuberculous mycobacterium after laser hair removal have been reported as serious complications [5,6]. A case of IE after electrolysis has also been reported [7]. These reports support the hypothesis that hair removal using laser or electrolysis may cause small abrasions that are invaded by the flora of skin bacteria such as *S. aureus*. In addition, *Staphylococcal* IE is mostly caused by frequent invasion of the bacteria through the skin, such as in cases of the insertion of intravascular devices or frequent intravenous injections [1]. It has also been reported that the incubation period from the time *S. aureus* enters the bloodstream to the onset of IE is about two weeks [8]. Therefore, we hypothesized that laser hair removal caused *S. aureus *to invade through the skin, leading to the development of *Staphylococcal* IE in our case.

To prevent the occurrence of IE, it is important to properly assess the risk for the development of IE. The potential risk of IE in young patients with native valves and a normal cardiac structure is generally extremely low. However, in our case, laser hair removal, in addition to the fragile skin barrier due to childhood atopic dermatitis, may have been the cause of bacteremia [9]. Whether laser hair removal is indeed the cause of bacterial invasion is not yet entirely clear, but patients with a fragile skin barrier may be at risk for bacteremia due to the laser. It is likely that neither the patients themselves nor their healthcare providers recognize laser hair removal as a risk for bacteremia. Therefore, it may be important to recognize the risk during esthetic procedures including laser hair removal, especially in populations with fragile skin barriers. In addition, recent increased concern for the sexual health and well-being of adults with congenital heart disease may increase the opportunities for laser hair removal to be performed on patients with a high risk for the development of IE [10]. If laser hair removal is a risk for bacteremia, as are dental procedures, the need for awareness for this high-risk IE population may be considered.

## Conclusions

Laser hair removal may cause *Staphylococcus aureus *bacteremia, which can lead to IE. Therefore, the potential association between laser hair removal and *Staphylococcal* IE should be recognized. Further investigation of the association between laser hair removal and bacteremia is needed to clarify this relationship. Exploring the possibility of bacteremia following laser hair removal may allow individuals at high risk for IE to be appropriately advised to discontinue laser hair removal. In addition, the association may provide valuable insights into the need for prevention of IE.
